# Successful Treatment of a Single Giant Renal Cyst in a Newborn with Drainage and Sclerotherapy

**DOI:** 10.1055/a-1939-4031

**Published:** 2022-11-11

**Authors:** Adriana Koenig, Anika Ménétrey, Tobias Jhala, Vincent Uerlings, Philipp O. Szavay

**Affiliations:** 1 Department of Pediatric Surgery, Luzerner Kantonsspital Kinderspital Luzern, Luzern, Switzerland; 2 Department of Pediatric Neurology, Universität Zürich, Kinderspital, Zurich, ZH, Switzerland; 3 Department of Gynaecology and Obstetrics, Luzerner Kantonsspital Kinderspital Luzern, Luzern, Switzerland

**Keywords:** renal cyst, sclerotherapy, single giant renal cyst

## Abstract

Simple renal cysts are a scarce entity in pediatric patients and their etiology is unknown in most cases. Usually, they are monitored with ultrasound and regular follow-up of renal function. Surgical treatment is rarely indicated. We report the case of a newborn with a single giant renal cyst that could be treated successfully with drainage and sclerotherapy. Single giant renal cysts require careful investigation and monitoring. In cysts without communication to the pelvico-caliceal system, sclerotherapy by instillation of doxycycline is a therapeutic option.

## Introduction

Simple renal cysts are a well-known entity in adults with a prevalence of more than 10% in patients above 50 years of age and over 30% in patients above 70 years of age. 1 Diagnosis is usually made by ultrasound and may be followed by computed tomography (CT) scan for better classification. Simple cysts appear as anechoic homogenous lesions with water content, sharp interface to the adjacent renal parenchyma and without wall thickening, calcifications, or distal enhancement. 2 To evaluate the risk of malignancy, the Bosniak system based on CT imaging is used for further classification. 2

In children, however, simple renal cysts are much less frequent with an incidence of 0.2 to 0.5%. 1 3 About 25% of these cysts are diagnosed prenatally or during the first year of life. 4 Most important differential diagnosis are hydronephrosis, renal neoplasms, 5 and polycystic kidney disease, especially the autosomal dominant form. 6 Other cystic diseases such as nephronophthisis, glomerulocystic kidney disease, or phakomatoses may be different rare causes for renal cysts. 1 3 The pathogenesis of simple renal cysts in children is unknown. Obstruction of renal tubules or caliceal diverticula is possible explanation that is yet to be proven. 5 Indications for treatment are pain, infection, hypertension, polycythemia, or obstruction of the collecting system. 7 When treatment is indicated, various options are available to diminish cystic volume. Percutaneous drainage under sonographic guidance, with or without sclerotherapy using different agents, as well as a surgical approach either open or laparoscopically for marsupialization, is possible choice. 5 7 8 9

In this report, we discuss the case of a newborn with a solitary giant renal cyst and its therapeutic approach.

## Case Report

Informed consent to publish patient history and images was obtained from the patient's mother.

At 30 weeks of gestational age, a single fluid-filled cyst in the right hemiabdomen was discovered, accompanied by a polyhydramnios. The cyst rapidly grew over the last few weeks of pregnancy that led to indication for a delivery of the baby boy prematurely at 36 4/7 gestational weeks. Birth weight was 3,320 g. Clinically, the baby presented with a distended abdomen with a palpable mass in the right hemiabdomen.

**Fig. 1:**
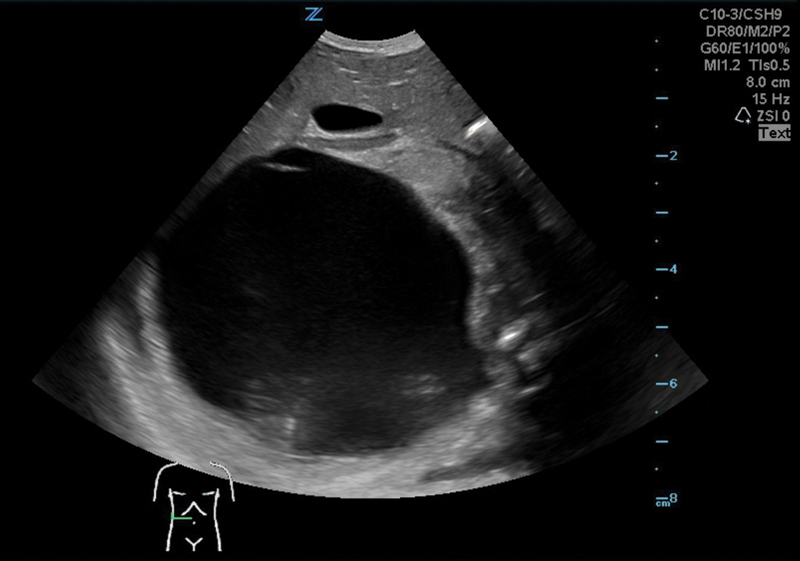
Right kidney at first day of life.

Ultrasound of the abdomen at first day of life showed a massive cyst (8.8 × 8 × 6.7 cm) in the right hemiabdomen without any signs of perfusion, distending the right kidney and adrenal gland. The left kidney appeared of normal morphology with minimally distended pyelon. The bowel was shifted to the left hemiabdomen, blood supply was not affected, and the liver appeared to be normal ( Fig. 1 ).

**Fig. 2:**
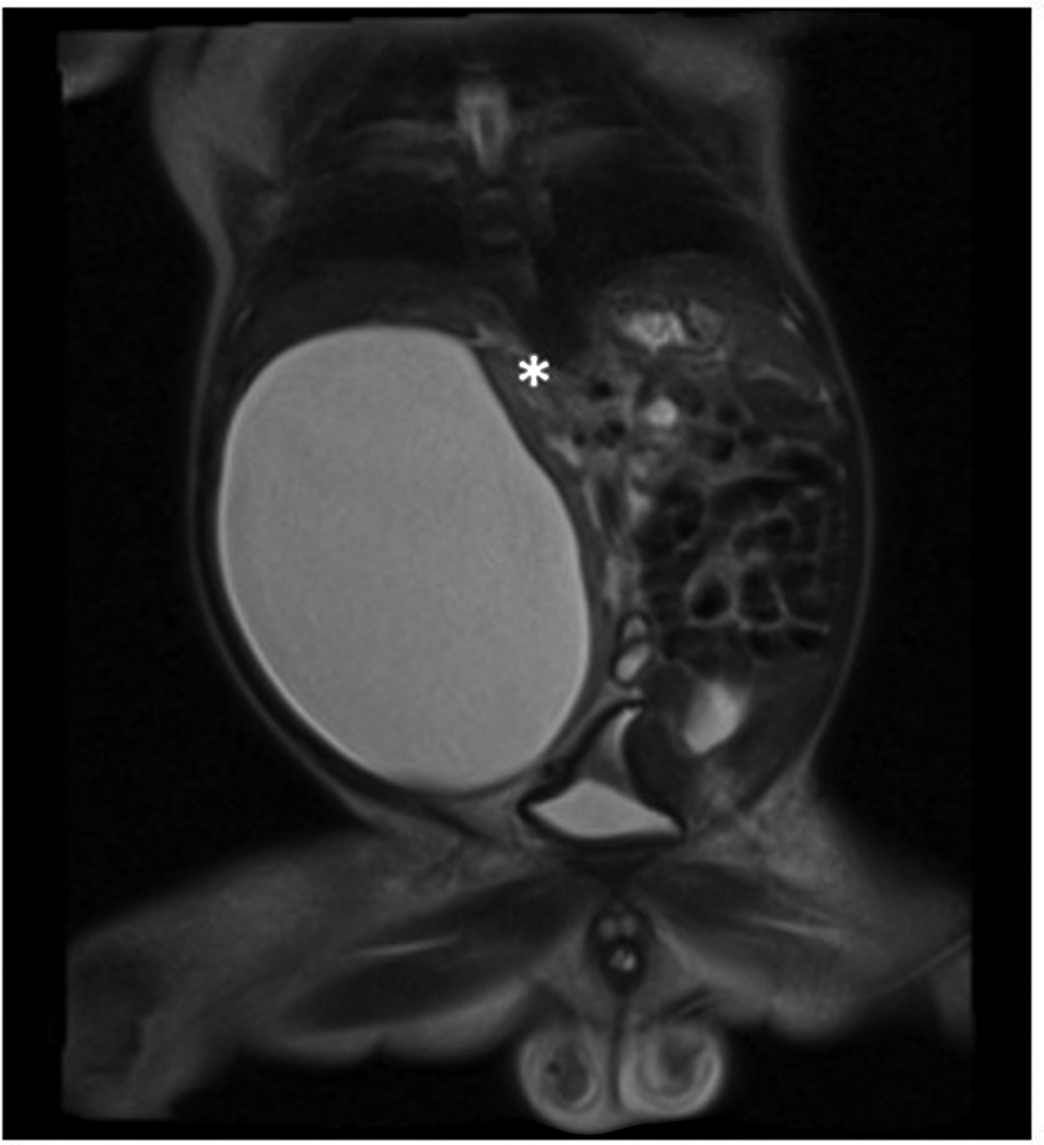
Magnetic resonance imaging at second day of life, asterisk indicating upper pole of right kidney.

For further clarification, a magnetic resonance imaging (MRI) was done at second day of life ( Fig. 2 ).

**Fig. 3:**
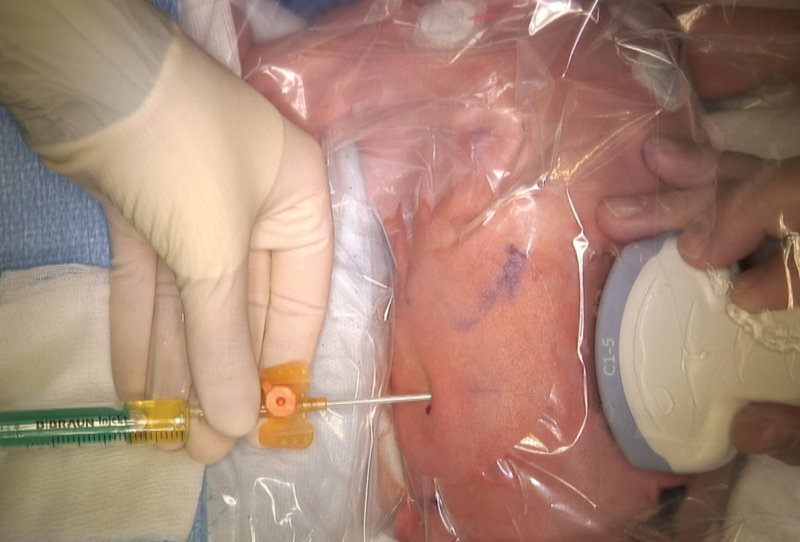
Pigtail insertion at third day of life.

MRI confirmed a renal origin of the cyst morphologically, in accordance with a Bosniak classification 1. Contrast was given, which did not appear in the cystic structure, thus excluding communication with the urinary tract collection system. Urinary drainage into the bladder was only slightly decelerated on the right and in normal range on the left side, hence any obstruction in the ureters or infravesically could be ruled out. The baby showed no signs of respiratory distress; however, feeding was not tolerated due to the mass effect of the cyst. For decompression and potential definitive treatment, sonographically guided drainage with insertion of a pigtail catheter was indicated ( Fig. 3 ) on the third day of life. We decided against laparoscopic intervention as initial treatment with percutaneous drainage was less invasive and promised good results with recurrence rates of 5% if sclerotherapy is performed as well. 5 Laparoscopy requires longer surgical time and published recurrence rates of simple renal cysts are 30 to 80% 7 which is why percutaneous drainage as first-line treatment was chosen. A clear yellow fluid (180 mL) could be drained that showed a creatinine level equal to serum level, thus confirming the absence of a functional connection to the pelvico-caliceal system as well. In addition, further findings of no bilirubin and a low amount of protein were consistent with a transudate. Cytological examinations did not reveal any malignant cells.

**Fig. 4:**
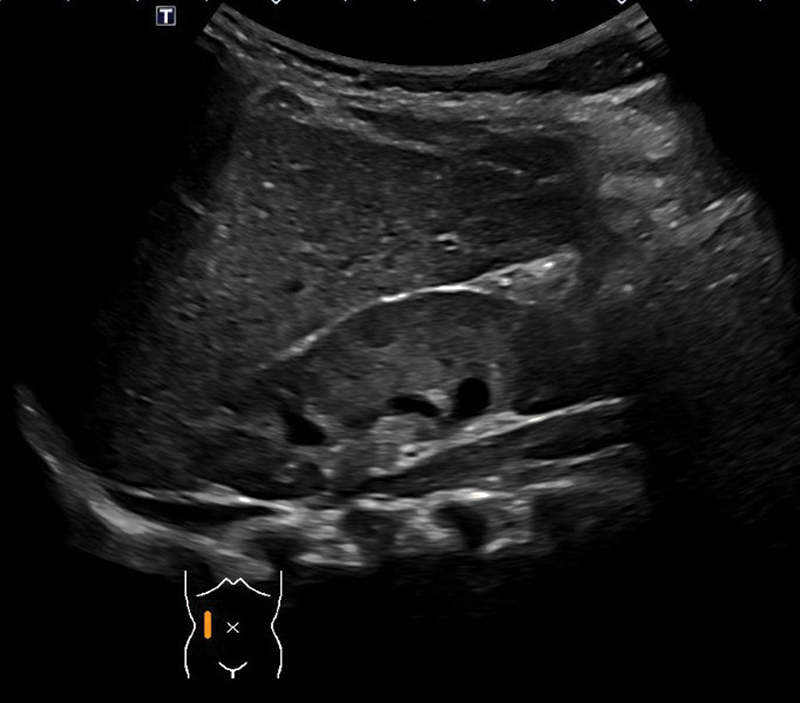
Ultrasound examination of right kidney at 1 year.

Blood creatinine levels, urine output, and liver function remained normal during hospitalization.

Following the intervention, the baby recovered quickly, and the pigtail catheter did not drain any more while ultrasound now showed a collapsed cyst. To induce chemical inflammation and consecutive obliteration of the cyst, indication for sclerotherapy was given. On postoperative day 6 (ninth day of life), 20 mg/kg doxycycline in a solution with normal saline to a total of 50ml was installed through the pigtail catheter which then was clamped. Doxycycline dosage was chosen according to guidelines concerning sclerotherapy for lymphatic malformations in children. 10 After a total dwelling time of 16 hours, the drain was opened. Following an initial bloody drainage of approximately 55 mL, fluid discharge ceased at postoperative day 8. Vitals, hemoglobin, and hematocrit values were stable during and after sclerotherapy. The pigtail catheter could then be removed on postoperative day 10. Ultrasound, thereafter, showed a cystic structure of 1.7 mL craniolateral to the right kidney. The patient was discharged on postoperative day 12.

Further outpatient follow-up was uneventful. Regular clinical and ultrasound exams were performed. Ultrasound at 1 year of age showed no evidence of the renal cyst with symmetrical renal parenchyma ( Fig. 4 ).

## Discussion Which Closes with a Conclusion

Simple renal cysts are rare in children with a frequency under 0.5%. Accordingly, reports of symptomatic simple renal cysts in newborns are scarce and in literature just one single case report of a newborn ( https://www.eurorad.org/case/9928 ) with a giant renal cyst that finally revealed to be a dysplastic kidney was found.

Simple renal cysts in childhood are usually managed conservatively and intervention is rarely necessary. On our patient, however, the enormous volume of the renal cyst caused a mass effect on the other internal organs, not only constricting the bowel, thereby urging us to intervene precociously. There is almost no literature on how to treat such a condition in a newborn. In case of normal renal function in asymptomatic patients, they are followed up with ultrasound. 2 In principle, drainage with or without instillation of a sclerotherapeutic agent as well as laparoscopic deroofing is possible treatment. Yet, laparoscopy in a newborn is an invasive measure, while recurrence rates are not insignificant. 7 In our case, sclerotherapy with a single instillation of doxycycline proved to be successful without any complications. Connection to the pelvico-caliceal system, however, would be considered a contraindication to perform any sclerotherapy. 10

In case of singular giant renal cyst in a newborn, sonographically-guided drainage and sclerotherapy with doxycycline are treatment options that might be successful.

## Notes

New Insights and Importance for a Pediatric Surgeon Simple renal cysts in children are rare. We report the case of a successful minimally invasive treatment.
